# (*E*)-*N*-[2-(3,5-Di-*tert*-butyl-2-hydroxy­benzyl­ideneamino)cyclo­hexyl]-4-methyl­benzene­sulfonamide

**DOI:** 10.1107/S1600536808043870

**Published:** 2009-01-08

**Authors:** Jincai Wu, Lei Wang, Xiaobo Pan, Lihui Yao

**Affiliations:** a College of Chemistry and Chemical Engineering, State Key Laboratory of Applied Organic Chemistry, Lanzhou University, Lanzhou 730000, People’s Republic of China

## Abstract

In the crystal structure of the title compound, C_28_H_40_N_2_O_3_S, there are two mol­ecules per asymmetric unit; in each of these mol­ecules, the cyclo­hexyl rings adopt chair conformations. The dihedral angles between the benzene rings are 16.89 (9) and 34.11 (9)°. Each mol­ecule contains an intra­molecular O—H⋯N hydrogen bond, and inter­molecular N—H⋯O hydrogen bonds are also present. In both mol­ecules, the methyl groups of one *tert*-butyl group are disordered over two positions; the site-occupancy factors in both cases are *ca* 0.6 and 0.4.

## Related literature

For the polymerization of cyclic esters, see: Endo *et al.* (1987 ▶); Wu *et al.* (2006 ▶). For synthetic details, see: Balsells *et al.* (1998 ▶).
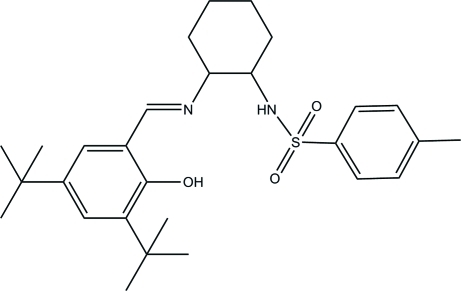


## Experimental

### 

#### Crystal data

C_28_H_40_N_2_O_3_S
*M* *_r_* = 484.68
Monoclinic, 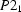

*a* = 10.3873 (10) Å
*b* = 25.773 (2) Å
*c* = 10.4127 (10) Å
β = 97.269 (2)°
*V* = 2765.2 (4) Å^3^
*Z* = 4
Mo *K*α radiation
μ = 0.15 mm^−1^
*T* = 298 (2) K
0.35 × 0.30 × 0.21 mm

#### Data collection

Bruker SMART 1K CCD diffractometer
Absorption correction: multi-scan (*MULscanABS* in *SADABS*; Sheldrick, 2002 ▶) *T*_min_ = 0.950, *T*_max_ = 0.970
15079 measured reflections
9696 independent reflections
7043 reflections with *I* > 2σ(*I*)
*R*_int_ = 0.034

#### Refinement

*R*[*F* ^2^ > 2σ(*F* ^2^)] = 0.045
*wR*(*F* ^2^) = 0.119
*S* = 0.97
9696 reflections
689 parameters
1 restraint
H-atom parameters constrained
Δρ_max_ = 0.26 e Å^−3^
Δρ_min_ = −0.25 e Å^−3^
Absolute structure: Flack (1983 ▶), 4173 Friedel pairs
Flack parameter: −0.04 (6)

### 

Data collection: *SMART* (Bruker, 2001 ▶); cell refinement: *SAINT* (Bruker, 2001 ▶); data reduction: *SAINT*; program(s) used to solve structure: *SHELXTL* (Sheldrick, 2008 ▶); program(s) used to refine structure: *SHELXTL*; molecular graphics: *SHELXTL*; software used to prepare material for publication: *SHELXTL*, *PLATON* (Spek, 2003 ▶) and local programs.

## Supplementary Material

Crystal structure: contains datablocks I, gobal. DOI: 10.1107/S1600536808043870/wn2301sup1.cif

Structure factors: contains datablocks I. DOI: 10.1107/S1600536808043870/wn2301Isup2.hkl

Additional supplementary materials:  crystallographic information; 3D view; checkCIF report

## Figures and Tables

**Table 1:** Hydrogen-bond geometry (Å, °)

*D*—H⋯*A*	*D*—H	H⋯*A*	*D*⋯*A*	*D*—H⋯*A*
O1—H1⋯N1	0.82	1.89	2.621 (3)	149
O4—H4*A*⋯N3	0.82	1.88	2.619 (3)	149
N2—H2*B*⋯O5^i^	0.86	2.36	2.987 (3)	130
N4—H4*B*⋯O2^ii^	0.86	2.30	2.950 (3)	133

Symmetry codes: (i) 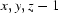; (ii) 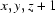.

**Fig. 1.**
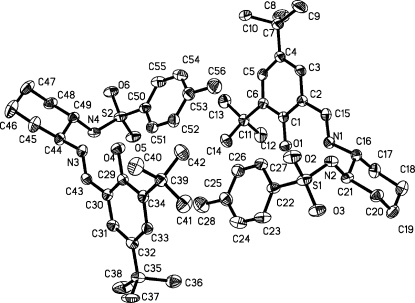
Molecular structure of the title compound. Displacement ellipsoids are drawn at the 30% probability level. H atoms have been omitted for clarity. The minor positions of the disordered tert-butyl groups are not shown.

**Fig. 2.**
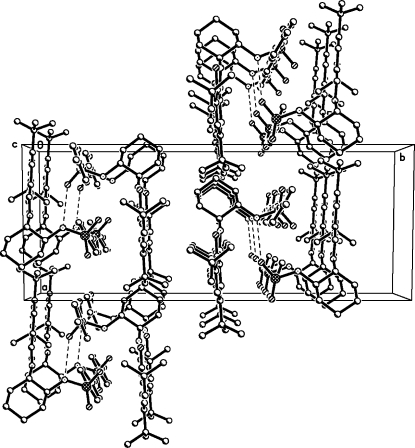
The packing of the title compound, viewed down the c axis, showing hydrogen bonds (dashed lines). H atoms have been omitted. The minor positions of the disordered tert-butyl groups are not shown.

## supplementary crystallographic information

### Comment

Recently, significant advances have been made in the polymerization of cyclic esters, such as poly-ε-caprolactone (Endo *et al.*, 1987), polylactide (Wu *et al.*, 2006) A particularly convenient method for the synthesis of polylactides is the ring-opening polymerization (ROP) of lactides. Due to the advantages of well-controlled molecular weight and low polydispersity (PDI), many metal complexes have been used. In the present study, we report a structure which is a potential ligand for the investigation of ROP of lactides.

As shown in Fig. 1, the asymmetric unit contains two molecules. In each of these molecules the cyclohexyl rings adopt chair conformations. The dihedral angles between the benzene rings are 16.89 (9)° and 34.11 (9)°. The bond lengths and angles are within normal ranges. Each molecule contains an intramolecular O—H···N hydrogen bond and intermolecular N—H···O hydrogen bonds are also present (Table 1 and Fig. 2). A short contact O2···.C43, with a distance of 3.011 (4) Å, involves the interaction of S=O and C=N.

### Experimental

The title compound was prepared as described in the literature (Balsells *et al.*, 1998). A fine crystal was obtained from an acetonitrile solution.

### Refinement

H atoms were placed in caculated positions and refined using a riding model, with d(N—H) = 0.86 Å and *U*_iso_(H) = 1.2U_eq_(N), d(O—H) = 0.82 Å and *U*_iso_(H) = 1.2U_eq(_O), d(C—H) = 0.93 Å and *U*_iso_(H) = 1.2U_eq_(C) for Csp^2^, d(C—H) = 0.97 Å and *U*_iso_(H) = 1.2U_eq_(C) for methylene groups, and d(C—H) = 0.96Å and *U*_iso_(H) = 1.5U_eq_(C) for methyl groups.

*PLATON* (Spek, 2003) suggests four A level alerts and two B level alerts which result from the disorder of *tert*-butyl groups. In both molecules the methyl groups of one tert-butyl group are disordered over two positions. The site occupancy factors for C8, C9, C10 and attached H atoms are 0.62 (2) and 0.38 (2); for C36, C37, C38 and attached H atoms the values are 0.621 (13) and 0.379 (13).

### Crystal data

**Table:**

C_28_H_40_N_2_O_3_S	*F*(000) = 1048
*M**_r_* = 484.68	*D*_x_ = 1.164 Mg m^−^^3^
Monoclinic, *P*2_1_	Mo *K*α radiation, λ = 0.71073 Å
Hall symbol: P 2yb	Cell parameters from 973 reflections
*a* = 10.3873 (10) Å	θ = 2.5–24.9°
*b* = 25.773 (2) Å	µ = 0.15 mm^−^^1^
*c* = 10.4127 (10) Å	*T* = 298 K
β = 97.269 (2)°	Block, yellow
*V* = 2765.2 (4) Å^3^	0.35 × 0.30 × 0.21 mm
*Z* = 4	

### Data collection

**Table:**

Bruker SMART 1K CCD diffractometer	9696 independent reflections
Radiation source: fine-focus sealed tube	7043 reflections with *I* > 2σ(*I*)
graphite	*R*_int_ = 0.034
φ and ω scans	θ_max_ = 26.0°, θ_min_ = 2.0°
Absorption correction: multi-scan (MULscanABS in *SADABS*; Sheldrick, 2002)	*h* = −12→9
*T*_min_ = 0.950, *T*_max_ = 0.970	*k* = −31→23
15079 measured reflections	*l* = −12→12

### Refinement

**Table:**

Refinement on *F*^2^	Hydrogen site location: inferred from neighbouring sites
Least-squares matrix: full	H-atom parameters constrained
*R*[*F*^2^ > 2σ(*F*^2^)] = 0.045	*w* = 1/[σ^2^(*F*_o_^2^) + (0.0663*P*)^2^] where *P* = (*F*_o_^2^ + 2*F*_c_^2^)/3
*wR*(*F*^2^) = 0.119	(Δ/σ)_max_ = 0.046
*S* = 0.97	Δρ_max_ = 0.26 e Å^−^^3^
9696 reflections	Δρ_min_ = −0.25 e Å^−^^3^
689 parameters	Extinction correction: *SHELXTL* (Sheldrick, 2008), Fc^*^=kFc[1+0.001xFc^2^λ^3^/sin(2θ)]^-1/4^
1 restraint	Extinction coefficient: 0.0038 (6)
Primary atom site location: structure-invariant direct methods	Absolute structure: Flack (1983), 4173 Friedel pairs
Secondary atom site location: difference Fourier map	Flack parameter: −0.04 (6)

### Special details

**Table:**

Geometry. All e.s.d.'s (except the e.s.d. in the dihedral angle between two l.s. planes) are estimated using the full covariance matrix. The cell e.s.d.'s are taken into account individually in the estimation of e.s.d.'s in distances, angles and torsion angles; correlations between e.s.d.'s in cell parameters are only used when they are defined by crystal symmetry. An approximate (isotropic) treatment of cell e.s.d.'s is used for estimating e.s.d.'s involving l.s. planes.
Refinement. Refinement of *F*^2^ against ALL reflections. The weighted *R*-factor *wR* and goodness of fit *S* are based on *F*^2^, conventional *R*-factors *R* are based on *F*, with *F* set to zero for negative *F*^2^. The threshold expression of *F*^2^ > σ(*F*^2^) is used only for calculating *R*-factors(gt) *etc*. and is not relevant to the choice of reflections for refinement. *R*-factors based on *F*^2^ are statistically about twice as large as those based on *F*, and *R*- factors based on ALL data will be even larger.

### Fractional atomic coordinates and isotropic or equivalent isotropic displacement parameters (Å^2^)

**Table:**

	*x*	*y*	*z*	*U*_iso_*/*U*_eq_	Occ. (<1)
S1	0.14819 (7)	0.14435 (3)	−0.18423 (8)	0.0499 (2)	
S2	0.58237 (7)	0.15336 (3)	0.83633 (8)	0.0513 (2)	
O1	0.29301 (19)	0.30301 (9)	0.1795 (2)	0.0526 (6)	
H1	0.2569	0.2997	0.1052	0.079*	
O2	0.2506 (2)	0.10829 (8)	−0.2013 (2)	0.0631 (6)	
O3	0.0211 (2)	0.13451 (8)	−0.2487 (2)	0.0621 (6)	
O4	0.42726 (19)	−0.00727 (9)	0.4676 (2)	0.0532 (6)	
H4A	0.4638	−0.0044	0.5418	0.080*	
O5	0.4811 (2)	0.18955 (8)	0.8563 (2)	0.0625 (6)	
O6	0.7097 (2)	0.16206 (10)	0.9016 (2)	0.0661 (7)	
N1	0.2698 (2)	0.29414 (10)	−0.0732 (2)	0.0432 (6)	
N2	0.1968 (2)	0.20004 (9)	−0.2292 (2)	0.0460 (6)	
H2B	0.2716	0.2031	−0.2554	0.055*	
N3	0.4524 (2)	0.00504 (9)	0.7192 (2)	0.0432 (6)	
N4	0.5323 (2)	0.09751 (10)	0.8777 (3)	0.0478 (6)	
H4B	0.4566	0.0949	0.9023	0.057*	
C1	0.4230 (3)	0.30549 (11)	0.1781 (3)	0.0395 (7)	
C6	0.5057 (3)	0.31103 (11)	0.2961 (3)	0.0410 (7)	
C11	0.4510 (3)	0.31494 (12)	0.4264 (3)	0.0461 (8)	
C14	0.3715 (4)	0.26723 (13)	0.4496 (3)	0.0611 (9)	
H14A	0.3409	0.2699	0.5325	0.092*	
H14B	0.4246	0.2368	0.4478	0.092*	
H14C	0.2988	0.2648	0.3831	0.092*	
C12	0.3668 (4)	0.36364 (15)	0.4286 (4)	0.0722 (11)	
H12A	0.3283	0.3645	0.5077	0.108*	
H12B	0.2996	0.3630	0.3563	0.108*	
H12C	0.4196	0.3939	0.4233	0.108*	
C13	0.5605 (4)	0.31917 (16)	0.5397 (3)	0.0707 (11)	
H13A	0.5238	0.3217	0.6196	0.106*	
H13B	0.6115	0.3496	0.5289	0.106*	
H13C	0.6147	0.2889	0.5416	0.106*	
C5	0.6368 (3)	0.31371 (12)	0.2871 (3)	0.0463 (7)	
H5B	0.6922	0.3180	0.3638	0.056*	
C4	0.6939 (3)	0.31053 (13)	0.1725 (3)	0.0475 (7)	
C7	0.8414 (3)	0.31449 (16)	0.1737 (4)	0.0644 (10)	
C8	0.8870 (9)	0.3671 (5)	0.2409 (16)	0.113 (4)	0.62 (2)
H8A	0.8671	0.3671	0.3285	0.169*	0.62 (2)
H8B	0.8429	0.3954	0.1943	0.169*	0.62 (2)
H8C	0.9790	0.3709	0.2410	0.169*	0.62 (2)
C9	0.8846 (12)	0.3123 (8)	0.0409 (11)	0.117 (7)	0.62 (2)
H9A	0.8514	0.2813	−0.0026	0.176*	0.62 (2)
H9B	0.9777	0.3121	0.0489	0.176*	0.62 (2)
H9C	0.8521	0.3421	−0.0082	0.176*	0.62 (2)
C10	0.9056 (11)	0.2708 (5)	0.2644 (12)	0.081 (3)	0.62 (2)
H10A	0.8815	0.2375	0.2277	0.121*	0.62 (2)
H10B	0.8763	0.2737	0.3480	0.121*	0.62 (2)
H10C	0.9983	0.2745	0.2733	0.121*	0.62 (2)
C9'	0.8752 (17)	0.3694 (8)	0.128 (5)	0.161 (17)	0.38 (2)
H9'A	0.8678	0.3939	0.1962	0.242*	0.38 (2)
H9'B	0.8162	0.3788	0.0531	0.242*	0.38 (2)
H9'C	0.9624	0.3696	0.1068	0.242*	0.38 (2)
C11'	0.8800 (17)	0.2763 (16)	0.065 (4)	0.155 (14)	0.38 (2)
H11A	0.9317	0.2484	0.1055	0.232*	0.38 (2)
H11B	0.9289	0.2949	0.0081	0.232*	0.38 (2)
H11C	0.8028	0.2624	0.0172	0.232*	0.38 (2)
C10'	0.927 (2)	0.302 (2)	0.293 (4)	0.21 (2)	0.38 (2)
H10D	0.9071	0.2683	0.3230	0.310*	0.38 (2)
H10E	0.9146	0.3274	0.3588	0.310*	0.38 (2)
H10F	1.0160	0.3032	0.2767	0.310*	0.38 (2)
C3	0.6092 (3)	0.30536 (13)	0.0596 (3)	0.0474 (7)	
H3C	0.6423	0.3039	−0.0192	0.057*	
C2	0.4751 (3)	0.30228 (11)	0.0611 (3)	0.0405 (7)	
C15	0.3927 (3)	0.29867 (12)	−0.0627 (3)	0.0436 (7)	
H15A	0.4321	0.2997	−0.1381	0.052*	
C16	0.1941 (3)	0.29483 (12)	−0.2025 (3)	0.0424 (7)	
H16A	0.2538	0.2968	−0.2681	0.051*	
C17	0.1084 (3)	0.34361 (13)	−0.2118 (3)	0.0585 (9)	
H17A	0.0605	0.3446	−0.1377	0.070*	
H17B	0.1638	0.3740	−0.2076	0.070*	
C18	0.0129 (4)	0.34630 (15)	−0.3347 (4)	0.0665 (10)	
H18A	0.0598	0.3506	−0.4088	0.080*	
H18B	−0.0437	0.3761	−0.3307	0.080*	
C19	−0.0682 (3)	0.29699 (15)	−0.3505 (3)	0.0596 (10)	
H19A	−0.1200	0.2940	−0.2797	0.072*	
H19B	−0.1266	0.2985	−0.4308	0.072*	
C20	0.0193 (3)	0.25048 (13)	−0.3517 (3)	0.0521 (8)	
H20A	0.0697	0.2534	−0.4237	0.063*	
H20B	−0.0333	0.2193	−0.3644	0.063*	
C21	0.1116 (3)	0.24572 (12)	−0.2249 (3)	0.0418 (7)	
H21A	0.0600	0.2416	−0.1530	0.050*	
C22	0.1358 (3)	0.14887 (13)	−0.0177 (3)	0.0453 (7)	
C23	0.0238 (3)	0.13469 (14)	0.0282 (4)	0.0661 (10)	
H23A	−0.0481	0.1246	−0.0287	0.079*	
C24	0.0178 (4)	0.13548 (16)	0.1614 (4)	0.0728 (11)	
H24A	−0.0578	0.1247	0.1925	0.087*	
C25	0.1218 (3)	0.15193 (14)	0.2483 (3)	0.0601 (9)	
C28	0.1151 (4)	0.15085 (19)	0.3919 (4)	0.0866 (13)	
H28A	0.1129	0.1155	0.4206	0.130*	
H28B	0.0382	0.1685	0.4103	0.130*	
H28C	0.1901	0.1679	0.4363	0.130*	
C26	0.2312 (3)	0.16790 (13)	0.1981 (4)	0.0584 (9)	
H26A	0.3014	0.1802	0.2542	0.070*	
C27	0.2404 (3)	0.16632 (13)	0.0671 (3)	0.0547 (9)	
H27A	0.3163	0.1769	0.0359	0.066*	
C29	0.2970 (3)	−0.00411 (11)	0.4683 (3)	0.0388 (6)	
C34	0.2129 (3)	−0.00827 (11)	0.3516 (3)	0.0399 (7)	
C39	0.2657 (3)	−0.01707 (13)	0.2213 (3)	0.0474 (8)	
C42	0.3567 (4)	0.02754 (16)	0.1953 (4)	0.0709 (11)	
H42A	0.4243	0.0307	0.2667	0.106*	
H42B	0.3083	0.0593	0.1853	0.106*	
H42C	0.3944	0.0205	0.1175	0.106*	
C40	0.3388 (4)	−0.06865 (15)	0.2242 (4)	0.0707 (11)	
H40A	0.4112	−0.0677	0.2914	0.106*	
H40B	0.3697	−0.0742	0.1422	0.106*	
H40C	0.2815	−0.0964	0.2408	0.106*	
C41	0.1576 (4)	−0.01910 (18)	0.1074 (3)	0.0726 (11)	
H41A	0.1117	0.0133	0.1011	0.109*	
H41B	0.0985	−0.0467	0.1207	0.109*	
H41C	0.1945	−0.0252	0.0288	0.109*	
C33	0.0807 (3)	−0.00399 (11)	0.3594 (3)	0.0415 (7)	
H33A	0.0243	−0.0061	0.2827	0.050*	
C32	0.0265 (3)	0.00322 (12)	0.4744 (3)	0.0437 (7)	
C35	−0.1219 (3)	0.00781 (13)	0.4743 (3)	0.0495 (8)	
C36	−0.1717 (6)	0.0548 (3)	0.3839 (10)	0.079 (3)	0.621 (13)
H36A	−0.1421	0.0507	0.3008	0.119*	0.621 (13)
H36B	−0.1384	0.0867	0.4225	0.119*	0.621 (13)
H36C	−0.2648	0.0556	0.3735	0.119*	0.621 (13)
C37	−0.1884 (10)	−0.0400 (3)	0.4122 (12)	0.083 (4)	0.621 (13)
H37A	−0.1703	−0.0428	0.3243	0.125*	0.621 (13)
H37B	−0.2804	−0.0371	0.4133	0.125*	0.621 (13)
H37C	−0.1567	−0.0703	0.4596	0.125*	0.621 (13)
C38	−0.1624 (7)	0.0177 (5)	0.6022 (7)	0.102 (5)	0.621 (13)
H38A	−0.1133	0.0461	0.6428	0.154*	0.621 (13)
H38B	−0.1474	−0.0128	0.6550	0.154*	0.621 (13)
H38C	−0.2531	0.0261	0.5924	0.154*	0.621 (13)
C36'	−0.1500 (12)	0.0580 (6)	0.529 (3)	0.132 (11)	0.379 (13)
H36D	−0.0879	0.0831	0.5077	0.199*	0.379 (13)
H36E	−0.1450	0.0550	0.6215	0.199*	0.379 (13)
H36F	−0.2357	0.0689	0.4942	0.199*	0.379 (13)
C37'	−0.1975 (16)	−0.0086 (14)	0.358 (2)	0.166 (16)	0.379 (13)
H37D	−0.1914	0.0167	0.2912	0.249*	0.379 (13)
H37E	−0.2865	−0.0123	0.3723	0.249*	0.379 (13)
H37F	−0.1656	−0.0414	0.3313	0.249*	0.379 (13)
C38'	−0.1498 (12)	−0.0362 (8)	0.5812 (18)	0.116 (7)	0.379 (13)
H38D	−0.0952	−0.0658	0.5732	0.174*	0.379 (13)
H38E	−0.2392	−0.0466	0.5662	0.174*	0.379 (13)
H38F	−0.1313	−0.0220	0.6667	0.174*	0.379 (13)
C31	0.1115 (3)	0.00599 (12)	0.5857 (3)	0.0447 (7)	
H31A	0.0788	0.0106	0.6640	0.054*	
C30	0.2460 (3)	0.00212 (11)	0.5860 (3)	0.0395 (7)	
C43	0.3298 (3)	0.00341 (12)	0.7095 (3)	0.0417 (7)	
H43A	0.2910	0.0030	0.7852	0.050*	
C44	0.5268 (3)	0.00251 (12)	0.8483 (3)	0.0460 (7)	
H44A	0.4660	0.0012	0.9129	0.055*	
C45	0.6056 (4)	−0.04741 (14)	0.8565 (3)	0.0651 (10)	
H45A	0.5466	−0.0767	0.8484	0.078*	
H45B	0.6561	−0.0487	0.7843	0.078*	
C46	0.6968 (4)	−0.05254 (17)	0.9822 (4)	0.0845 (13)	
H46A	0.7492	−0.0836	0.9795	0.101*	
H46B	0.6468	−0.0556	1.0544	0.101*	
C47	0.7842 (4)	−0.00538 (18)	1.0010 (4)	0.0784 (12)	
H47A	0.8384	−0.0037	0.9318	0.094*	
H47B	0.8405	−0.0083	1.0825	0.094*	
C48	0.7029 (3)	0.04415 (14)	1.0013 (3)	0.0602 (9)	
H48A	0.6514	0.0429	1.0726	0.072*	
H48B	0.7602	0.0740	1.0140	0.072*	
C49	0.6135 (3)	0.05031 (12)	0.8744 (3)	0.0441 (7)	
H49A	0.6670	0.0539	0.8038	0.053*	
C50	0.5933 (3)	0.15180 (12)	0.6684 (3)	0.0465 (7)	
C51	0.4966 (3)	0.12880 (13)	0.5829 (3)	0.0562 (9)	
H51A	0.4270	0.1124	0.6142	0.067*	
C52	0.5036 (3)	0.13026 (12)	0.4533 (3)	0.0572 (9)	
H52A	0.4391	0.1142	0.3971	0.069*	
C53	0.6054 (3)	0.15531 (13)	0.4025 (3)	0.0571 (9)	
C56	0.6095 (4)	0.15820 (17)	0.2584 (4)	0.0808 (12)	
H56A	0.5600	0.1301	0.2166	0.121*	
H56B	0.6979	0.1557	0.2410	0.121*	
H56C	0.5733	0.1906	0.2260	0.121*	
C54	0.7014 (4)	0.17739 (15)	0.4897 (4)	0.0667 (10)	
H54A	0.7706	0.1941	0.4587	0.080*	
C55	0.6973 (3)	0.17529 (14)	0.6205 (4)	0.0619 (9)	
H55B	0.7643	0.1896	0.6772	0.074*	

### Atomic displacement parameters (Å^2^)

**Table:**

	*U*^11^	*U*^22^	*U*^33^	*U*^12^	*U*^13^	*U*^23^
S1	0.0363 (4)	0.0466 (5)	0.0653 (5)	−0.0005 (4)	0.0010 (3)	−0.0134 (4)
S2	0.0368 (4)	0.0521 (5)	0.0641 (5)	−0.0009 (4)	0.0024 (4)	−0.0160 (4)
O1	0.0394 (12)	0.0753 (16)	0.0429 (13)	0.0017 (11)	0.0049 (10)	−0.0054 (11)
O2	0.0485 (13)	0.0521 (14)	0.0887 (18)	0.0055 (11)	0.0090 (13)	−0.0206 (12)
O3	0.0440 (12)	0.0625 (16)	0.0764 (16)	−0.0099 (11)	−0.0051 (11)	−0.0178 (12)
O4	0.0415 (12)	0.0774 (17)	0.0407 (12)	0.0045 (11)	0.0058 (10)	−0.0053 (11)
O5	0.0462 (13)	0.0517 (14)	0.0903 (18)	0.0060 (10)	0.0108 (12)	−0.0237 (12)
O6	0.0416 (13)	0.0784 (18)	0.0746 (17)	−0.0114 (12)	−0.0065 (12)	−0.0147 (13)
N1	0.0471 (15)	0.0451 (15)	0.0368 (14)	−0.0050 (12)	0.0031 (12)	−0.0030 (11)
N2	0.0363 (14)	0.0494 (15)	0.0532 (16)	−0.0010 (11)	0.0095 (12)	−0.0073 (12)
N3	0.0499 (15)	0.0450 (15)	0.0332 (13)	0.0024 (12)	−0.0011 (11)	−0.0015 (11)
N4	0.0357 (14)	0.0532 (16)	0.0558 (17)	0.0023 (12)	0.0112 (12)	−0.0057 (12)
C1	0.0435 (17)	0.0359 (16)	0.0394 (17)	0.0038 (13)	0.0065 (13)	−0.0016 (13)
C6	0.0452 (17)	0.0394 (16)	0.0384 (17)	0.0022 (13)	0.0055 (14)	−0.0043 (13)
C11	0.058 (2)	0.0478 (18)	0.0331 (17)	0.0068 (15)	0.0099 (15)	−0.0057 (14)
C14	0.079 (2)	0.056 (2)	0.052 (2)	0.0010 (18)	0.0240 (19)	0.0016 (16)
C12	0.096 (3)	0.063 (2)	0.061 (2)	0.026 (2)	0.022 (2)	−0.0090 (17)
C13	0.080 (3)	0.092 (3)	0.039 (2)	0.002 (2)	0.0022 (19)	−0.0076 (18)
C5	0.0507 (19)	0.0474 (18)	0.0388 (17)	0.0019 (14)	−0.0019 (14)	−0.0020 (13)
C4	0.0438 (17)	0.0572 (19)	0.0418 (18)	−0.0043 (14)	0.0066 (14)	0.0051 (14)
C7	0.0435 (19)	0.096 (3)	0.052 (2)	−0.0048 (19)	−0.0003 (16)	0.012 (2)
C8	0.062 (5)	0.120 (8)	0.153 (11)	−0.037 (5)	0.003 (6)	0.022 (8)
C9	0.068 (6)	0.22 (2)	0.065 (6)	−0.019 (9)	0.025 (4)	0.020 (8)
C10	0.038 (5)	0.122 (8)	0.085 (6)	0.016 (5)	0.017 (4)	0.031 (6)
C9'	0.075 (11)	0.108 (14)	0.30 (5)	−0.029 (9)	0.031 (17)	0.07 (2)
C11'	0.030 (7)	0.22 (3)	0.22 (3)	−0.024 (12)	0.043 (12)	−0.11 (2)
C10'	0.063 (11)	0.33 (5)	0.22 (3)	−0.03 (2)	0.012 (15)	0.18 (4)
C3	0.0489 (18)	0.061 (2)	0.0339 (16)	−0.0021 (15)	0.0126 (14)	0.0005 (14)
C2	0.0453 (17)	0.0381 (16)	0.0380 (17)	−0.0025 (13)	0.0042 (13)	−0.0006 (13)
C15	0.0537 (19)	0.0456 (18)	0.0317 (17)	−0.0005 (15)	0.0067 (14)	−0.0061 (13)
C16	0.0458 (18)	0.0502 (18)	0.0312 (17)	−0.0010 (14)	0.0050 (14)	−0.0022 (13)
C17	0.072 (2)	0.050 (2)	0.051 (2)	0.0014 (17)	−0.0022 (18)	−0.0002 (16)
C18	0.070 (2)	0.066 (2)	0.061 (2)	0.0103 (19)	−0.0034 (19)	0.0070 (18)
C19	0.048 (2)	0.074 (2)	0.054 (2)	0.0076 (18)	−0.0077 (17)	0.0013 (18)
C20	0.0451 (17)	0.063 (2)	0.0460 (18)	−0.0020 (16)	−0.0036 (15)	−0.0072 (15)
C21	0.0334 (15)	0.0510 (18)	0.0409 (17)	0.0027 (13)	0.0040 (13)	−0.0086 (13)
C22	0.0360 (15)	0.0349 (16)	0.065 (2)	−0.0026 (14)	0.0046 (14)	−0.0021 (15)
C23	0.0415 (18)	0.076 (3)	0.081 (3)	−0.0112 (17)	0.0072 (18)	−0.013 (2)
C24	0.052 (2)	0.083 (3)	0.087 (3)	−0.0130 (19)	0.026 (2)	−0.007 (2)
C25	0.069 (2)	0.0467 (19)	0.066 (2)	−0.0021 (19)	0.0150 (18)	0.0020 (17)
C28	0.101 (3)	0.086 (3)	0.076 (3)	−0.016 (3)	0.024 (2)	−0.007 (2)
C26	0.058 (2)	0.052 (2)	0.063 (2)	−0.0067 (16)	0.0020 (18)	0.0050 (16)
C27	0.0438 (18)	0.055 (2)	0.065 (2)	−0.0095 (15)	0.0066 (17)	0.0055 (16)
C29	0.0415 (16)	0.0391 (16)	0.0360 (16)	0.0002 (13)	0.0059 (13)	−0.0005 (12)
C34	0.0482 (17)	0.0420 (17)	0.0300 (15)	0.0048 (13)	0.0065 (13)	0.0004 (12)
C39	0.0511 (18)	0.056 (2)	0.0359 (17)	0.0052 (16)	0.0091 (14)	−0.0007 (14)
C42	0.083 (3)	0.080 (3)	0.054 (2)	−0.006 (2)	0.025 (2)	0.0034 (19)
C40	0.088 (3)	0.072 (3)	0.053 (2)	0.023 (2)	0.014 (2)	−0.0115 (17)
C41	0.078 (2)	0.105 (3)	0.0347 (19)	0.015 (2)	0.0072 (18)	−0.0123 (19)
C33	0.0435 (16)	0.0413 (17)	0.0391 (17)	−0.0018 (13)	0.0030 (13)	0.0016 (13)
C32	0.0462 (17)	0.0445 (17)	0.0418 (18)	−0.0028 (14)	0.0112 (15)	−0.0055 (14)
C35	0.0392 (16)	0.054 (2)	0.057 (2)	−0.0060 (15)	0.0119 (15)	−0.0111 (16)
C36	0.055 (4)	0.067 (5)	0.118 (7)	0.011 (3)	0.018 (4)	0.007 (4)
C37	0.048 (5)	0.068 (5)	0.136 (11)	−0.017 (4)	0.019 (6)	−0.011 (4)
C38	0.048 (4)	0.204 (16)	0.061 (5)	0.005 (6)	0.026 (3)	−0.030 (6)
C36'	0.044 (7)	0.093 (11)	0.26 (3)	0.002 (7)	0.035 (11)	−0.033 (14)
C37'	0.036 (7)	0.36 (5)	0.098 (15)	0.034 (18)	−0.021 (9)	−0.09 (2)
C38'	0.054 (7)	0.138 (16)	0.160 (16)	−0.027 (8)	0.029 (8)	0.044 (12)
C31	0.0484 (18)	0.0528 (19)	0.0343 (17)	−0.0032 (15)	0.0109 (14)	−0.0045 (13)
C30	0.0429 (16)	0.0403 (16)	0.0347 (15)	0.0025 (13)	0.0029 (13)	−0.0035 (12)
C43	0.0470 (18)	0.0421 (17)	0.0375 (18)	0.0000 (14)	0.0106 (14)	0.0014 (13)
C44	0.0504 (18)	0.0566 (19)	0.0301 (15)	0.0046 (15)	0.0019 (13)	0.0008 (13)
C45	0.079 (2)	0.061 (2)	0.051 (2)	0.0168 (19)	−0.0068 (19)	0.0011 (16)
C46	0.096 (3)	0.089 (3)	0.063 (3)	0.034 (3)	−0.015 (2)	0.012 (2)
C47	0.060 (2)	0.112 (4)	0.059 (2)	0.032 (2)	−0.0091 (19)	0.006 (2)
C48	0.0456 (18)	0.082 (3)	0.050 (2)	0.0018 (18)	−0.0049 (16)	−0.0044 (17)
C49	0.0389 (15)	0.0540 (19)	0.0393 (17)	0.0085 (14)	0.0048 (13)	−0.0025 (14)
C50	0.0380 (15)	0.0366 (16)	0.064 (2)	0.0059 (15)	0.0043 (15)	−0.0039 (15)
C51	0.0469 (18)	0.052 (2)	0.066 (2)	−0.0078 (15)	−0.0040 (17)	0.0070 (16)
C52	0.060 (2)	0.051 (2)	0.056 (2)	−0.0075 (16)	−0.0080 (18)	0.0048 (16)
C53	0.063 (2)	0.0436 (19)	0.066 (2)	0.0089 (17)	0.0131 (18)	0.0031 (17)
C56	0.088 (3)	0.079 (3)	0.078 (3)	0.005 (2)	0.020 (2)	0.006 (2)
C54	0.054 (2)	0.065 (2)	0.084 (3)	−0.0067 (18)	0.023 (2)	−0.003 (2)
C55	0.0429 (19)	0.062 (2)	0.081 (3)	−0.0047 (16)	0.0079 (18)	−0.0157 (19)

### Geometric parameters (Å, °)

**Table:**

S1—O3	1.426 (2)	C24—C25	1.385 (5)
S1—O2	1.441 (2)	C24—H24A	0.9300
S1—N2	1.610 (3)	C25—C26	1.373 (5)
S1—C22	1.759 (3)	C25—C28	1.505 (5)
S2—O6	1.426 (2)	C28—H28A	0.9600
S2—O5	1.441 (2)	C28—H28B	0.9600
S2—N4	1.608 (3)	C28—H28C	0.9600
S2—C50	1.767 (3)	C26—C27	1.380 (5)
O1—C1	1.354 (3)	C26—H26A	0.9300
O1—H1	0.8200	C27—H27A	0.9300
O4—C29	1.357 (3)	C29—C30	1.404 (4)
O4—H4A	0.8200	C29—C34	1.408 (4)
N1—C15	1.274 (4)	C34—C33	1.390 (4)
N1—C16	1.471 (4)	C34—C39	1.543 (4)
N2—C21	1.477 (4)	C39—C41	1.528 (4)
N2—H2B	0.8600	C39—C40	1.529 (5)
N3—C43	1.265 (4)	C39—C42	1.534 (5)
N3—C44	1.464 (4)	C42—H42A	0.9600
N4—C49	1.483 (4)	C42—H42B	0.9600
N4—H4B	0.8600	C42—H42C	0.9600
C1—C2	1.396 (4)	C40—H40A	0.9600
C1—C6	1.415 (4)	C40—H40B	0.9600
C6—C5	1.379 (4)	C40—H40C	0.9600
C6—C11	1.538 (4)	C41—H41A	0.9600
C11—C14	1.518 (4)	C41—H41B	0.9600
C11—C12	1.532 (4)	C41—H41C	0.9600
C11—C13	1.535 (4)	C33—C32	1.399 (4)
C14—H14A	0.9600	C33—H33A	0.9300
C14—H14B	0.9600	C32—C31	1.366 (4)
C14—H14C	0.9600	C32—C35	1.546 (4)
C12—H12A	0.9600	C35—C37'	1.424 (17)
C12—H12B	0.9600	C35—C36'	1.458 (15)
C12—H12C	0.9600	C35—C38	1.469 (7)
C13—H13A	0.9600	C35—C37	1.517 (10)
C13—H13B	0.9600	C35—C36	1.580 (8)
C13—H13C	0.9600	C35—C38'	1.641 (14)
C5—C4	1.400 (4)	C36—H36A	0.9600
C5—H5B	0.9300	C36—H36B	0.9600
C4—C3	1.382 (4)	C36—H36C	0.9600
C4—C7	1.535 (4)	C37—H37A	0.9600
C7—C10'	1.47 (3)	C37—H37B	0.9600
C7—C9	1.508 (12)	C37—H37C	0.9600
C7—C9'	1.548 (19)	C38—H38A	0.9600
C7—C10	1.563 (13)	C38—H38B	0.9600
C7—C8	1.570 (12)	C38—H38C	0.9600
C7—C11'	1.59 (2)	C36'—H36D	0.9600
C8—H8A	0.9600	C36'—H36E	0.9600
C8—H8B	0.9600	C36'—H36F	0.9600
C8—H8C	0.9600	C37'—H37D	0.9600
C9—H9A	0.9600	C37'—H37E	0.9600
C9—H9B	0.9600	C37'—H37F	0.9600
C9—H9C	0.9600	C38'—H38D	0.9600
C10—H10A	0.9600	C38'—H38E	0.9600
C10—H10B	0.9600	C38'—H38F	0.9600
C10—H10C	0.9600	C31—C30	1.401 (4)
C9'—H9'A	0.9600	C31—H31A	0.9300
C9'—H9'B	0.9600	C30—C43	1.460 (4)
C9'—H9'C	0.9600	C43—H43A	0.9300
C11'—H11A	0.9600	C44—C49	1.530 (4)
C11'—H11B	0.9600	C44—C45	1.521 (4)
C11'—H11C	0.9600	C44—H44A	0.9800
C10'—H10D	0.9600	C45—C46	1.522 (5)
C10'—H10E	0.9600	C45—H45A	0.9700
C10'—H10F	0.9600	C45—H45B	0.9700
C3—C2	1.397 (4)	C46—C47	1.515 (6)
C3—H3C	0.9300	C46—H46A	0.9700
C2—C15	1.458 (4)	C46—H46B	0.9700
C15—H15A	0.9300	C47—C48	1.531 (5)
C16—C21	1.530 (4)	C47—H47A	0.9700
C16—C17	1.537 (4)	C47—H47B	0.9700
C16—H16A	0.9800	C48—C49	1.524 (4)
C17—C18	1.518 (4)	C48—H48A	0.9700
C17—H17A	0.9700	C48—H48B	0.9700
C17—H17B	0.9700	C49—H49A	0.9800
C18—C19	1.522 (5)	C50—C51	1.388 (4)
C18—H18A	0.9700	C50—C55	1.385 (5)
C18—H18B	0.9700	C51—C52	1.362 (5)
C19—C20	1.505 (5)	C51—H51A	0.9300
C19—H19A	0.9700	C52—C53	1.398 (5)
C19—H19B	0.9700	C52—H52A	0.9300
C20—C21	1.536 (4)	C53—C54	1.384 (5)
C20—H20A	0.9700	C53—C56	1.508 (5)
C20—H20B	0.9700	C56—H56A	0.9600
C21—H21A	0.9800	C56—H56B	0.9600
C22—C23	1.363 (4)	C56—H56C	0.9600
C22—C27	1.385 (4)	C54—C55	1.369 (5)
C23—C24	1.396 (5)	C54—H54A	0.9300
C23—H23A	0.9300	C55—H55B	0.9300
			
O3—S1—O2	118.76 (13)	H28A—C28—H28B	109.5
O3—S1—N2	109.09 (14)	C25—C28—H28C	109.5
O2—S1—N2	106.00 (13)	H28A—C28—H28C	109.5
O3—S1—C22	107.31 (14)	H28B—C28—H28C	109.5
O2—S1—C22	108.15 (14)	C25—C26—C27	122.1 (3)
N2—S1—C22	106.99 (14)	C25—C26—H26A	118.9
O6—S2—O5	118.66 (14)	C27—C26—H26A	118.9
O6—S2—N4	108.97 (15)	C26—C27—C22	119.5 (3)
O5—S2—N4	105.89 (14)	C26—C27—H27A	120.2
O6—S2—C50	107.67 (14)	C22—C27—H27A	120.2
O5—S2—C50	107.28 (14)	O4—C29—C30	119.9 (2)
N4—S2—C50	107.95 (14)	O4—C29—C34	120.1 (2)
C1—O1—H1	109.5	C30—C29—C34	120.1 (3)
C29—O4—H4A	109.5	C33—C34—C29	116.9 (3)
C15—N1—C16	119.4 (3)	C33—C34—C39	121.8 (3)
C21—N2—S1	119.41 (19)	C29—C34—C39	121.3 (3)
C21—N2—H2B	120.3	C41—C39—C40	107.5 (3)
S1—N2—H2B	120.3	C41—C39—C42	107.3 (3)
C43—N3—C44	118.7 (3)	C40—C39—C42	109.8 (3)
C49—N4—S2	121.58 (19)	C41—C39—C34	112.3 (2)
C49—N4—H4B	119.2	C40—C39—C34	110.0 (3)
S2—N4—H4B	119.2	C42—C39—C34	109.9 (3)
O1—C1—C2	120.2 (3)	C39—C42—H42A	109.5
O1—C1—C6	119.5 (2)	C39—C42—H42B	109.5
C2—C1—C6	120.3 (3)	H42A—C42—H42B	109.5
C5—C6—C1	116.2 (3)	C39—C42—H42C	109.5
C5—C6—C11	122.3 (3)	H42A—C42—H42C	109.5
C1—C6—C11	121.4 (3)	H42B—C42—H42C	109.5
C14—C11—C12	109.8 (3)	C39—C40—H40A	109.5
C14—C11—C13	107.5 (3)	C39—C40—H40B	109.5
C12—C11—C13	107.4 (3)	H40A—C40—H40B	109.5
C14—C11—C6	110.9 (2)	C39—C40—H40C	109.5
C12—C11—C6	110.0 (3)	H40A—C40—H40C	109.5
C13—C11—C6	111.2 (3)	H40B—C40—H40C	109.5
C11—C14—H14A	109.5	C39—C41—H41A	109.5
C11—C14—H14B	109.5	C39—C41—H41B	109.5
H14A—C14—H14B	109.5	H41A—C41—H41B	109.5
C11—C14—H14C	109.5	C39—C41—H41C	109.5
H14A—C14—H14C	109.5	H41A—C41—H41C	109.5
H14B—C14—H14C	109.5	H41B—C41—H41C	109.5
C11—C12—H12A	109.5	C34—C33—C32	124.6 (3)
C11—C12—H12B	109.5	C34—C33—H33A	117.7
H12A—C12—H12B	109.5	C32—C33—H33A	117.7
C11—C12—H12C	109.5	C31—C32—C33	116.5 (3)
H12A—C12—H12C	109.5	C31—C32—C35	122.3 (3)
H12B—C12—H12C	109.5	C33—C32—C35	121.2 (3)
C11—C13—H13A	109.5	C37'—C35—C36'	118.8 (14)
C11—C13—H13B	109.5	C37'—C35—C38	129.3 (9)
H13A—C13—H13B	109.5	C36'—C35—C38	52.9 (9)
C11—C13—H13C	109.5	C36'—C35—C37	141.6 (7)
H13A—C13—H13C	109.5	C38—C35—C37	111.1 (7)
H13B—C13—H13C	109.5	C37'—C35—C32	114.9 (8)
C6—C5—C4	125.7 (3)	C36'—C35—C32	108.5 (5)
C6—C5—H5B	117.1	C38—C35—C32	114.4 (4)
C4—C5—H5B	117.1	C37—C35—C32	109.8 (5)
C3—C4—C5	116.0 (3)	C37'—C35—C36	67.5 (15)
C3—C4—C7	122.7 (3)	C36'—C35—C36	59.1 (10)
C5—C4—C7	121.3 (3)	C38—C35—C36	107.3 (6)
C10'—C7—C9	123.4 (15)	C37—C35—C36	105.7 (5)
C10'—C7—C4	119.3 (10)	C32—C35—C36	108.1 (3)
C9—C7—C4	113.7 (6)	C37'—C35—C38'	104.3 (15)
C10'—C7—C9'	108.6 (14)	C36'—C35—C38'	106.6 (12)
C9—C7—C9'	69.4 (14)	C38—C35—C38'	53.7 (8)
C4—C7—C9'	108.8 (7)	C37—C35—C38'	67.5 (8)
C9—C7—C10	111.9 (8)	C32—C35—C38'	102.0 (6)
C4—C7—C10	107.8 (5)	C36—C35—C38'	149.5 (6)
C9'—C7—C10	138.5 (11)	C35—C36—H36A	109.5
C10'—C7—C8	72 (2)	C35—C36—H36B	109.5
C9—C7—C8	109.5 (7)	C35—C36—H36C	109.5
C4—C7—C8	107.8 (5)	C35—C37—H37A	109.5
C10—C7—C8	105.7 (7)	C35—C37—H37B	109.5
C10'—C7—C11'	107 (2)	C35—C37—H37C	109.5
C4—C7—C11'	107.1 (7)	C35—C38—H38A	109.5
C9'—C7—C11'	105.2 (14)	C35—C38—H38B	109.5
C10—C7—C11'	81.7 (18)	C35—C38—H38C	109.5
C8—C7—C11'	139.7 (10)	C35—C36'—H36D	109.5
C7—C8—H8A	109.5	C35—C36'—H36E	109.5
C7—C8—H8B	109.5	H36D—C36'—H36E	109.5
C7—C8—H8C	109.5	C35—C36'—H36F	109.5
C7—C9—H9A	109.5	H36D—C36'—H36F	109.5
C7—C9—H9B	109.4	H36E—C36'—H36F	109.5
C7—C9—H9C	109.5	C35—C37'—H37D	109.5
C7—C10—H10A	109.5	C35—C37'—H37E	109.5
C7—C10—H10B	109.5	H37D—C37'—H37E	109.5
C7—C10—H10C	109.5	C35—C37'—H37F	109.5
C7—C9'—H9'A	109.4	H37D—C37'—H37F	109.5
C7—C9'—H9'B	109.5	H37E—C37'—H37F	109.5
H9'A—C9'—H9'B	109.5	C35—C38'—H38D	109.5
C7—C9'—H9'C	109.5	C35—C38'—H38E	109.5
H9'A—C9'—H9'C	109.5	H38D—C38'—H38E	109.5
H9'B—C9'—H9'C	109.5	C35—C38'—H38F	109.5
C7—C11'—H11A	109.5	H38D—C38'—H38F	109.5
C7—C11'—H11B	109.4	H38E—C38'—H38F	109.5
H11A—C11'—H11B	109.5	C32—C31—C30	122.4 (3)
C7—C11'—H11C	109.4	C32—C31—H31A	118.8
H11A—C11'—H11C	109.5	C30—C31—H31A	118.8
H11B—C11'—H11C	109.5	C31—C30—C29	119.5 (3)
C7—C10'—H10D	109.4	C31—C30—C43	118.9 (3)
C7—C10'—H10E	109.5	C29—C30—C43	121.5 (3)
H10D—C10'—H10E	109.5	N3—C43—C30	123.6 (3)
C7—C10'—H10F	109.5	N3—C43—H43A	118.2
H10D—C10'—H10F	109.5	C30—C43—H43A	118.2
H10E—C10'—H10F	109.5	N3—C44—C49	111.0 (2)
C4—C3—C2	121.6 (3)	N3—C44—C45	107.8 (2)
C4—C3—H3C	119.2	C49—C44—C45	111.7 (3)
C2—C3—H3C	119.2	N3—C44—H44A	108.7
C3—C2—C1	120.2 (3)	C49—C44—H44A	108.7
C3—C2—C15	117.9 (3)	C45—C44—H44A	108.7
C1—C2—C15	121.8 (3)	C46—C45—C44	113.2 (3)
N1—C15—C2	123.5 (3)	C46—C45—H45A	108.9
N1—C15—H15A	118.3	C44—C45—H45A	108.9
C2—C15—H15A	118.3	C46—C45—H45B	108.9
N1—C16—C21	110.7 (2)	C44—C45—H45B	108.9
N1—C16—C17	107.8 (2)	H45A—C45—H45B	107.8
C21—C16—C17	111.0 (2)	C45—C46—C47	109.8 (3)
N1—C16—H16A	109.1	C45—C46—H46A	109.7
C21—C16—H16A	109.1	C47—C46—H46A	109.7
C17—C16—H16A	109.1	C45—C46—H46B	109.7
C18—C17—C16	113.7 (3)	C47—C46—H46B	109.7
C18—C17—H17A	108.8	H46A—C46—H46B	108.2
C16—C17—H17A	108.8	C46—C47—C48	110.4 (3)
C18—C17—H17B	108.8	C46—C47—H47A	109.6
C16—C17—H17B	108.8	C48—C47—H47A	109.6
H17A—C17—H17B	107.7	C46—C47—H47B	109.6
C17—C18—C19	110.3 (3)	C48—C47—H47B	109.6
C17—C18—H18A	109.6	H47A—C47—H47B	108.1
C19—C18—H18A	109.6	C49—C48—C47	111.2 (3)
C17—C18—H18B	109.6	C49—C48—H48A	109.4
C19—C18—H18B	109.6	C47—C48—H48A	109.4
H18A—C18—H18B	108.1	C49—C48—H48B	109.4
C20—C19—C18	109.9 (3)	C47—C48—H48B	109.4
C20—C19—H19A	109.7	H48A—C48—H48B	108.0
C18—C19—H19A	109.7	N4—C49—C48	110.6 (2)
C20—C19—H19B	109.7	N4—C49—C44	109.9 (2)
C18—C19—H19B	109.7	C48—C49—C44	110.6 (3)
H19A—C19—H19B	108.2	N4—C49—H49A	108.6
C19—C20—C21	111.6 (3)	C48—C49—H49A	108.6
C19—C20—H20A	109.3	C44—C49—H49A	108.6
C21—C20—H20A	109.3	C51—C50—C55	119.4 (3)
C19—C20—H20B	109.3	C51—C50—S2	120.8 (2)
C21—C20—H20B	109.3	C55—C50—S2	119.8 (3)
H20A—C20—H20B	108.0	C52—C51—C50	119.9 (3)
N2—C21—C16	109.8 (2)	C52—C51—H51A	120.1
N2—C21—C20	110.4 (2)	C50—C51—H51A	120.1
C16—C21—C20	110.0 (2)	C51—C52—C53	121.7 (3)
N2—C21—H21A	108.9	C51—C52—H52A	119.1
C16—C21—H21A	108.9	C53—C52—H52A	119.1
C20—C21—H21A	108.9	C54—C53—C52	117.3 (3)
C23—C22—C27	120.0 (3)	C54—C53—C56	121.6 (3)
C23—C22—S1	120.0 (2)	C52—C53—C56	121.1 (3)
C27—C22—S1	120.0 (2)	C53—C56—H56A	109.5
C22—C23—C24	119.4 (3)	C53—C56—H56B	109.5
C22—C23—H23A	120.3	H56A—C56—H56B	109.5
C24—C23—H23A	120.3	C53—C56—H56C	109.5
C25—C24—C23	121.7 (3)	H56A—C56—H56C	109.5
C25—C24—H24A	119.2	H56B—C56—H56C	109.5
C23—C24—H24A	119.2	C55—C54—C53	121.7 (3)
C26—C25—C24	117.2 (3)	C55—C54—H54A	119.1
C26—C25—C28	121.8 (3)	C53—C54—H54A	119.1
C24—C25—C28	120.9 (3)	C54—C55—C50	120.0 (3)
C25—C28—H28A	109.5	C54—C55—H55B	120.0
C25—C28—H28B	109.5	C50—C55—H55B	120.0

### Hydrogen-bond geometry (Å, °)

**Table:**

*D*—H···*A*	*D*—H	H···*A*	*D*···*A*	*D*—H···*A*
O1—H1···N1	0.82	1.89	2.621 (3)	149
O4—H4A···N3	0.82	1.88	2.619 (3)	149
N2—H2B···O5^i^	0.86	2.36	2.987 (3)	130
N4—H4B···O2^ii^	0.86	2.30	2.950 (3)	133

Symmetry codes: (i) *x*, *y*, *z*−1; (ii) *x*, *y*, *z*+1.
